# Impairment of Vitamin D Nutritional Status and Metabolic Profile Are Associated with Worsening of Obesity According to the Edmonton Obesity Staging System

**DOI:** 10.3390/ijms232314705

**Published:** 2022-11-25

**Authors:** Adryana Cordeiro, Mariana Luna, Silvia Elaine Pereira, Carlos José Saboya, Andrea Ramalho

**Affiliations:** 1 Micronutrients Research Center (NPqM), Department of Social Applied Nutrition, Institute of Nutrition, Federal University of Rio de Janeiro (UFRJ), Rio de Janeiro 21941-901, Brazil; 2 Multidisciplinary Center of Bariatric and Metabolic Surgery Carlos Saboya, Rio de Janeiro 21941-902, Brazil

**Keywords:** obesity, vitamin D, Edmonton Obesity Staging System, metabolic diseases, non-communicable chronic diseases

## Abstract

Obesity is associated with a higher risk of Vitamin D (VD) inadequacy and metabolic diseases. The Edmonton Obesity Staging System (EOSS) is an innovative tool for the evaluation of obesity that goes beyond body weight and considers clinic, functional and menta- health issues. This study aimed to evaluate the nutritional status of VD according to the stages of EOSS and its relationship with the metabolic profile. In the cross-sectional study, we evaluated anthropometric parameters, physical activity, blood pressure, biochemical and metabolic variables, and VD nutritional status. A total of 226 individuals were categorized using EOSS: 1.3%, 22.1%, 62.9%, and 13.7% were in stages 0, 1, 2 and 3, respectively. Regarding the metabolic changes and comorbidities, insulin resistance and hyperuricemia were diagnosed in some individuals in EOSS 1, 2, and 3. EOSS 2 and 3 presented a significant relative-risk for the development of arterial hypertension, metabolic syndrome, and liver disease, compared with EOSS 0. In all stages, there were observed means of 25(OH)D serum concentrations below 30 ng/mL (EOSS 0 24.9 ± 3.3 ng/mL; EOSS 3 15.9 ± 5.4 ng/mL; *p* = 0.031), and 25(OH)D deficiency was present in all stages. Individuals with obesity classified in more advanced stages of EOSS had lower serum concentrations of 25(OH)D and a worse metabolic profile.

## 1. Introduction

Obesity is considered an important public health problem worldwide [1]. In the last four decades, its prevalence has tripled, currently affecting more than 650 million individuals, approximately 13% of the world population [2]. In Brazil, 17.7% of the population are affected by the disease [3]. It is extremely worrying, as obesity is considered one of the main risk factors for non-communicable chronic diseases (NCDs) such as cardiovascular diseases, type 2 diabetes mellitus (T2DM), metabolic-associated fatty liver disease (MAFLD) and cancer. Thus, its impacts include reducing quality of life and increasing morbidity and mortality [2,4].

In addition, obesity is also related to multiple micronutrient deficiencies, which contribute to worse individual health status [5,6]. In this context, Vitamin D (VD) stands out as having an inverse correlation with high body-adiposity. The discovery of the vitamin D nuclear receptor (VDR), ubiquitously expressed in almost all cells of the body, including immune, vascular, and myocardial cells, has suggested the implication of vitamin D-mediated effects in several systems beyond musculoskeletal tissues, such as chronic diseases [7]. Low concentrations of 25-hydroxyvitamin D [25(OH)D] are observed in individuals with obesity [8]. Some mechanisms could explain it, such as: (i) lower synthesis of 25(OH)D in the liver is a cause of steatosis; (ii) VD could be stored in adipose tissue, decreasing its bioavailability and resulting in a worsening cycle that might reduce circulating VD levels; (iii) serum 25(OH)D is directly related to lipoprotein lipase, an enzyme that is involved in fat synthesis; (iv) vitamin D would be involved in energy metabolism and adipocyte biology through the regulation of β-oxidation [9].

Regarding the assessment and classification of obesity, currently, the most used parameter in clinical practice is the body mass index (BMI), often combined with other anthropometric measures, such as waist circumference (WC) [10]. However, the use of BMI individually and in isolation does not allow the assessment of body composition [11]. Even when combined with some anthropometric parameters, information on the presence of important factors for optimizing the clinical assessment is not considered [4].

Aiming to minimize the limitations of the obesity classification parameters most used in clinical practice, the “Edmonton Obesity Staging System” (EOSS) was developed, which is an ordinal staging system with five degrees of severity, based on a simple clinical analysis. This system considers the presence and severity of risk factors, comorbidities, functional limitations, and quality of life of the individual, proposing a deeper and more detailed assessment of obesity and the impact of excess body fat on the health and well-being of the individual [4].

Therefore, the aim of this study is to evaluate the nutritional status of VD according to the stages of EOSS and its relationship with metabolic profile, in individuals with obesity classified by this classification system.

## 2. Results

In the present study, 226 individuals with severe obesity participated. Of these, 1.3% (*n* = 3), 22.1% (*n* = 50), 62.9% (*n* = 142) and 13.7% (*n* = 31) were classified as 0, 1, 2 and 3 of the EOSS, respectively. Individuals were not categorized for stage 4.

The stages did not differ in relation to BMI, with the average for EOSS 0: 40.3 ± 5.7 kg/m^2^, *p* = 0.421; EOSS 1: 42.4 ± 3.4 kg/m^2^, *p* = 0.770; EOSS 2: 43.7 ± 3.9 kg/m^2^, *p* = 0.554; EOSS 3: 44.1 ± 1.2 kg/m^2^, *p* = 0.553.

When physical activity was assessed, 78%, 50%, 30%, and 16% performed some type of physical exercise such as low-intensity gym, walking or stretching (between 15 to 30 min, 2–3 times a week), in EOSS 0, 1, 2 and 3, respectively.

Regarding metabolic changes and comorbidities, insulin resistance (IR) and hyperuricemia were diagnosed in some individuals in stages of EOSS (1 to 3), without significant difference between the groups. In addition, arterial hypertension (AH) was observed in individuals classified in stages 2 and 3. It is also worth mentioning that in the individuals classified in EOSS 1, 7.6% were diagnosed with MS and 17.1% with hypertriglyceridemia (Table 1). 

**Figure 1:**
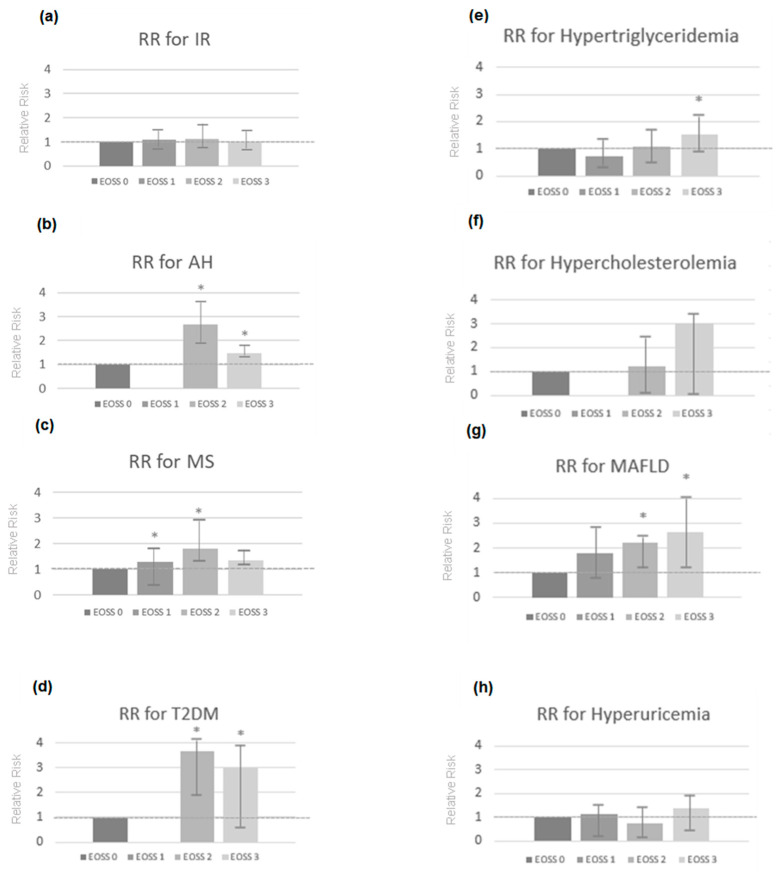
Association between Edmonton Obesity Staging System (EOSS) stage and risk of IR (**a**), AH (**b**), MS (**c**), T2DM (**d**), hypertriglyceridemia (**e**), hypercholesterolemia (**f**), MAFLD (**g**) and hyperuricemia (**h**) in the sample. * *p* ≤ 0.05. IR, insulin resistance; AH, arterial hypertension; MS, metabolic syndrome; T2DM, type 2 diabetes mellitus; MAFLD, metabolic-associated fatty liver disease; RR, relative risk. Cox proportional hazards regression.

EOSS 2 and 3 presented a significant relative risk (RR) for the development of AH (EOSS 2–RR (95% CI): 2.57 (1.89–3.14) *p* = 0.030; EOSS 3–RR: 1.64 (1.13–1.76) *p* ≤ 0.001); SM (EOSS 2–RR: 1.85 (1.42–2.32), *p* ≤ 0.001; EOSS 3–RR: 1.36 (1.16–1.169) *p* = 0.007); T2DM (EOSS 2–RR: 3.68 (1.73–3.82), *p* ≤ 0.001; EOSS 3–RR: 2.98 (0.48–3.99) *p* = 0.019); hypertriglyceridemia (EOSS 3–RR: 1.88 (1.22–2.75) *p* = 0.039) and MAFLD (EOSS 2–RR: 2.22 (1.11–2.41) *p* = 0.004; EOSS 3–RR: 2.62 (1.31–3.70) *p* ≤ 0.001) compared with EOSS 0. Individuals who were at EOSS 1 had a RR for developing MS (EOSS 1–RR (95% CI): 1.29 (0.17–1.47), *p* ≤ 0.001) (Figure 1).

Regarding the presence of MAFLD, 3 individuals had SS (simple steatosis) (EOSS 1); 16 had SS and 3 had nonalcoholic steatohepatitis (NASH) without fibrosis (EOSS 2); and 12 had SS, 14 had NASH without fibrosis and 2 had NASH with fibrosis (EOSS 3).

The means of hs-CRP according to the stages of EOSS were 0.8 ± 0.6 mg/dL, 0.9 ± 0.3 mg/dL, 1.3 ± 0.7 mg/dL and 2.6 ± 0.5 mg/dL; *p* = 0.034, in EOSS 0, 1, 2 and 3, respectively.

**Figure 2:**
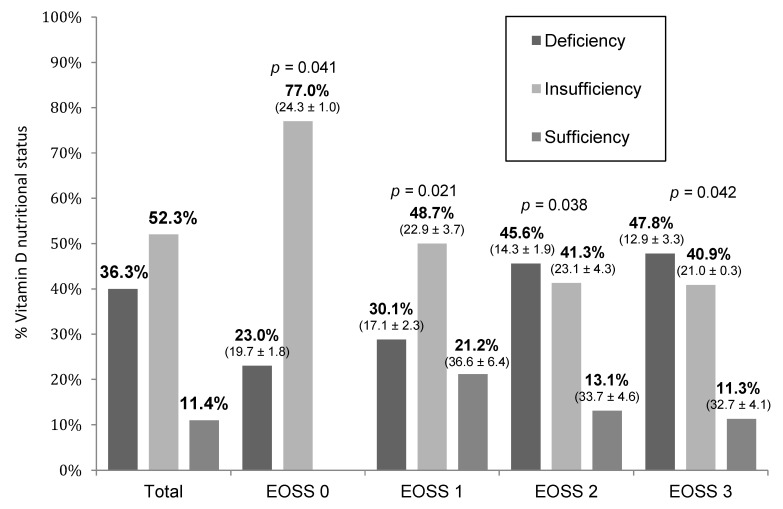
Percentage and mean/standard deviation (ng/mL) of serum concentrations of 25(OH)D for each nutritional status of Vitamin D according to the stage of the Edmonton Obesity Staging System (EOSS). ANOVA. EOSS: Edmonton Obesity Staging System.

For the nutritional status of Vitamin D, this study observed an 88.6% prevalence of inadequacy (36.3% of deficiency and 52.3% of insufficiency). The means of 25(OH)D serum concentrations below 30 ng/mL were observed (EOSS 0 = 24.9 ± 3.3 ng/mL; EOSS 1 = 22.4 ± 5.6 ng/mL; EOSS 2 = 18.7 ± 6.7 ng/mL; EOSS 3 = 15.9 ± 5.4 ng/mL; *p* = 0.031), and 25(OH)D deficiency was presented in all stages of EOSS (Figure 2).

When assessing the mean serum concentrations of VD in the presence of IR, individuals in EOSS 3 had a lower mean of 25(OH)D (13.8 ± 2.5 ng/mL) and those in EOSS 1 presented a higher mean (21.7 ± 3.4 ng/mL), with a significant difference between groups (*p* = 0.022).

Among the metabolic changes and comorbidities analyzed, MS was associated with the nutritional status of VD in stages 1, 2, and 3 of the EOSS (*p* ≤ 0.001; *p* ≤ 0.001, *p* = 0.043, respectively). There was no significant association between hypercholesterolemia with the nutritional status of VD in the different stages of EOSS.

## 3. Discussion

The present study has an originality, since it is the first to demonstrate the applicability of an innovative method for classifying obesity which is aligned with the multifactorial aspects of this disease and related to the nutritional status of VD and the metabolic profile. Among the main findings is that a lower serum concentration of 25(OH)D and a higher prevalence of metabolic alterations are observed in individuals with obesity, according to the progression in the EOSS stages, even in the absence of anthropometric differences them. Furthermore, the presence of metabolic heterogeneity, even in the face of anthropometric similarity, highlights the importance of classifying obesity by more complete methods, and not only by anthropometric criteria.

Currently, obesity care has been mostly restricted to anthropometric parameters, such as BMI and WC, indirect indicators of body-fat accumulation. These parameters are used both for the assessment and staging of obesity and as eligibility criteria for some interventions, such as bariatric surgery. However, it is increasingly perceived that they have limitations in their ability to fully assess the individual with obesity, since they reflect only the total accumulation of body fat, for example, and not the degree of health impairment and quality of life caused by excess adiposity.

The EOSS stands out for using variables related to the complications of obesity as criteria for staging, proposing a detailed evaluation, considering clinical parameters, mental health, functional impairment, and quality of life. These are evaluated gradually; that is, the greater the severity of the metabolic manifestations, the more advanced the classification stage.

Recent evidence suggests that EOSS may be a determining factor in the success of interventions for the treatment and control of obesity, and in public health costs. As the stages of EOSS progress, less weight loss and less metabolic improvement emerge, both after bariatric surgery and after conventional multidisciplinary treatment, and the use of health services is greater [12,13,14].

The literature shows that an inverse correlation between BMI and 25(OH)D is increasingly established, so that, the greater the increase in this anthropometric parameter, the greater the impairment of VD serum concentrations [15,16]. In the present study, the results demonstrate that the more advanced the classification of obesity by EOSS, the more inadequate the nutritional status of VD, suggesting the ability of this classification system also to indicate relevant information about it. Thus, the classification of obesity performed by the EOSS system is in accordance with what has been observed in the scientific literature, reinforcing its applicability to clinical practice.

The present study observed a higher mean serum-concentration of VD in stage 0 of the EOSS and a higher percentage of individuals with sufficiency. In contrast, in EOSS 3, a lower average of 25(OH)D was obtained, with statistical significance. Possible explanations for the relationship between obesity and impairment of vitamin D nutritional-status are based on excess of adiposity, increased body volume, impaired hepatic 25-hydroxylation [17], the influence of low-grade chronic inflammation noted in this condition, differential gene expression of vitamin D metabolizing-enzymes, and the poor lifestyle to which people find themselves subjected, such as decreased sun exposure and inadequate diet.

In addition, when considering the classification of EOSS, the nutritional status of VD, and the comorbidities studied, the presence of lower concentrations of 25(OH)D was observed in more advanced stages of EOSS, as well as greater metabolic impairment. Conditions such as IR and low-grade inflammation can contribute to these findings; VD could influence the insulin secretion regulated by the opening and closing of calcium channels and 1,25(OH)2D, the active form of VD, may also upgrade insulin sensitivity by stimulating the expression of insulin receptors and activating the peroxisome proliferator-activated receptor delta (PPAR-δ) [18].

According to evidence from humans and animals, both obesity and VD deficiency seem to be strongly correlated with changes in insulin sensitivity, which can be considered the main predictor of metabolic changes. The dysregulation of insulin signaling pathways is the main factor that causes the reduction in its sensitivity and the installation of IR, the most frequent cause of T2DM, and is directly associated with MS and MAFLD [19].

The proposed mechanisms by which VD influences glycemic homeostasis also involve the modulation of insulin synthesis/secretion mediated by glucose and pancreatic β cells, increasing hepatic and peripheral glucose uptake by direct and indirect mechanisms. This is because 1,25(OH)2D acts as a chemical messenger, by interacting with calcium flux-regulating receptors on the β-cell [18]. In addition, the development of IR in adipocytes is related to impaired insulin signaling, and the decreased insulin receptor substrate 1 (IRS1) expression and increased insulin receptor substrate 2 (IRS2), which are key substrates for Phosphoinositide 3-kinases (PI3K), a family of enzymes involved in cellular functions, were found in IR state in adipocytes [20].

Another important point is the inverse correlation between inflammatory markers and 25(OH)D serum concentrations observed in recent studies [21,22,23]. Moreover, insulin-resistant adipocytes present decreased expression of GLUT4, the insulin-regulated glucose transporter, and alterations in the profile of secreted adipocytokines such as leptin, tumor necrosis factor α (TNF-α), and adiponectin [20]. This suggests a negative impact of inflammation on the nutritional status of VD.

In addition, mechanisms have been proposed for the beneficial effects of VD supplementation on CRP concentrations; it has been suggested that this vitamin regulates the production of macrophage-related inflammatory cytokines through calcium-dependent mechanisms. VD may decrease inflammation via the regulation of serum concentrations of calcium and the suppression of parathyroid hormone (PTH) and circulating levels of PTH are positively associated with serum levels of inflammatory biomarkers [24].

Thus, the inflammatory state of obesity may be one of the main explanations for the increase in the inadequacy of the nutritional status of VD, together with a higher prevalence of metabolic changes, such as the progression of EOSS stages. In view of the fact that IR and the presence of inflammation contribute to the development of such changes, it can be assumed that individuals categorized in higher stages of EOSS have a more exacerbated degree of inflammation. Consequently, this inflammatory state may be contributing to the reduction of 25(OH)D serum concentrations, which is a possible justification for the results obtained. As VD has anti-inflammatory effects, modulates insulin-sensitivity, local inflammation, and adipokine secretion, a supplementation of VD could be useful in improving islet-cell function, insulin release, IR [25], adipose tissue oxidative-stress [26] and local concentrations of pro-inflammatory cytokines.

The strength of this study was to demonstrate the applicability of a more complete obesity classification method and its relationship with the metabolic profile and nutritional status of VD. However, the present study has the limitation of using a cross-sectional model that does not allow causal relationships to be established.

## 4. Materials and Methods

A cross-sectional study comprising 226 individuals with obesity (body mass index (BMI) ≥ 35 kg/m^2^), aged ≥ 20 and <60 years and in the preoperative phase of bariatric surgery, recruited from the patients of a medical clinic specializing in the control of obesity, in Rio de Janeiro, Brazil, from November 2018 to July 2019, and conducted according to CONSORT guidelines. Exclusion criteria were as follows: acute or chronic infections, elevated serum-calcium levels, pregnancy or lactation, history and/or presence of chronic kidney (defined by estimated GFR < 60 mL/min/1.73 m^2^) [27] or liver diseases (except nonalcoholic fatty liver disease), malabsorption bowel syndrome, previous restrictive and disabsorptive surgeries, diagnosis of endocrinopathies (hyperparathyroidism, hypothyroidism, hypercortisolemia), use of anticonvulsant medications or drugs known to interfere with vitamin D metabolism, as well as current insulin treatment and the consumption of vitamin D supplements within 6 months prior to the blood test. This study was approved by the Research Ethics Committee of Hospital Universitário Clementino Fraga Filho, Federal University of Rio de Janeiro, Brazil (Research Protocol number 011/06-CEP).

All patients were informed that participation in the study was voluntary. Written informed consent was obtained before carrying out any study-related procedures from all subjects who participated in the study.

### 4.1. Sample Size

The sample size was determined to respond to the main aim of the study, which was to evaluate the nutritional status of VD according to the stages of EOSS. The following parameters have been assumed: use of bilateral tests, a level of significance of 5%, a statistical power of 80%, and an expected correlation of −0.25.

According to the sample calculation, 224 individuals were required. The sample-size value was inflated by 10%, to anticipate possible losses.

### 4.2. Evaluation of Anthropometric Parameters and Physical Activity

The BMI calculation (kg/m^2^) was conducted based on the anthropometric measurements of weight (kg) and height (m) [28].

Waist/height ratio (WHtR) was calculated using the formula: WC (cm)/height (m). The cutoff point was 0.50, in line with Zeng Q et al. [29].

Data related to the habit of engaging in physical exercise, such as type, time (in years and minutes/week) and weekly frequency (days/week) were collected through a questionnaire previously prepared [30] during the first consultation.

### 4.3. Evaluation of Clinical, Biochemical and Metabolic Parameters

The blood pressure quantification by an indirect measurement method was carried out using the OMRON HEM-705CP monitor (OMRON Healthcare Europe B.V., Hoofddorp, The Netherlands), with a range of 0–300 mmHg and an accuracy of ±3 mmHg. At least two measurements were taken, with an interval of approximately one minute, and the mean was calculated.

For biochemical and metabolic evaluation, blood was obtained by venipuncture, after an overnight fasting period of 12 h. Laboratory tests were conducted in serum, to characterize the lipid profile (total cholesterol, low-density lipoprotein cholesterol (LDL-c), high-density lipoprotein cholesterol (HDL-c) and triglycerides) and to evaluate the glucose, hemoglobin A1c (HbA1c), insulin, Homeostatic model assessment—insulin resistance (HOMA-IR), homeostatic model assessment of β-cell function (HOMA β), uric acid and high sensitivity- C Reactive Protein (hs-CRP) levels.

Determinations of total cholesterol, HDL-c, triglycerides, and glucose were performed using specific enzymatic colorimetric-methods (Labtest Diagnóstica S.A., Minas Gerais, Brazil). LDL-c fraction was calculated in accordance with Friedewald’s formula [31]. HbA1c measurements were performed using high-performance liquid chromatography (HPLC), (HumaNexA1c system, Human, Wiesbaden, Germany). Insulin was quantified using reversed-phase HPLC (Labtest Diagnóstica S.A., Minas Gerais, Brazil).

HOMA-IR and HOMA-β were calculated using the Homeostasis Model Assessment (HOMA2) Calculator v2.2, which is available from the Oxford Centre for Diabetes, Endocrinology, and Metabolism [32].

### 4.4. Diagnosis of Metabolic Syndrome (MS)

The criteria proposed by the Third Report of the National Cholesterol Education Program Expert Panel on Detection, Evaluation, and Treatment of High Blood Cholesterol in Adults (NCEP-ATP III) were used for the evaluation and diagnostic of MS. Individuals who had alterations in ≥3 of the following 5 criteria were diagnosed with MS: (1) WC > 102 cm for men and > 88 cm for women; (2) fasting glucose ≥ 100 mg/dL; (3) fasting triglycerides ≥ 150 mg/dL; (4) HDL-c < 40 mg/dL for men and < 50 mg/dL for women; and (5) blood pressure ≥ 130/ ≥ 85 mmHg.

### 4.5. Diagnosis of MAFLD—Liver Biopsy

Histological evaluation was conducted through a withdrawal of 4 mm thickness of the left lobe of the liver via puncture, using a 16 G × 15 cm Menghini needle (Euromed, Minas Gerais, Brazil). Biopsies were conducted by the medical surgeon along with the bariatric surgery.

Grading was performed considering the presence of macrovesicular steatosis (SS) and necroinflammatory activity (presence of NASH).

#### 4.5.1. Macrovesicular Steatosis

Grade 0: no steatosis; Grade 1 (mild): <33% of fat accumulation in hepatocytes; Grade 2 (moderate): between 33% and 66% of hepatocytes affected; Grade 3 (severe): >66% of hepatocytes affected.

#### 4.5.2. Necroinflammatory Activity (NASH)

Grade 0: no steatosis; Grade 1: mild; Grade 2: moderate; Grade 3: severe.

Staging of Fibrosis Was Performed in Individuals with NASH

Stage 0: no steatosis; Stage 1: presence of pericellular or perisinusoidal fibrosis in zone 3, focal or extensive; Stage 2: presence of pericellular or perisinusoidal fibrosis in zone 3 associated with the presence of focal or extensive periportal fibrosis; Stage 3: presence of pericellular or perisinusoidal fibrosis in zone 3 and focal or extensive fibrosis bridges; Stage 4: cirrhosis.

After a systematic sampling technique in which, for each five patients evaluated during the study period, one was selected for the stage of MAFLD by liver biopsy, a subsample of 50 individuals was selected.

### 4.6. Vitamin D Status

Serum vitamin D analysis was conducted in the form of 25(OH)D using HPLC with UV detector (Chromsystems, Bio Sys Ltd., Rio de Janeiro, Brazil). The cutoff points used were deficiency (≤20 ng/mL), insufficiency (20.1 ng/mL–29.9 ng/mL) or sufficiency (≥30 ng/mL and <100 ng/mL) group [33]. To complete the evaluation of the nutritional status of vitamin D, an investigation was conducted into the exposure to the sun of the individuals, as described by Hanwell et al. [34].

### 4.7. Edmonton Obesity Staging System (EOSS)

All information on metabolic risk factors, medication usage, and self-reported doctor diagnosis of obesity-related morbidities was extracted from electronic patient files. We categorized EOSS staging by using the highest-stage risk factor for each patient, based on modified operational definitions adapted from Sharma and Kushner 4, displayed in Table 2. For example, a patient with obesity-related subclinical risk factors (borderline high glucose, borderline high blood-pressure, etc.), mild physical symptoms, mild psychopathology, and mild functional limitations (stage 1) but diagnosed with arthritis (stage 2) would be categorized as EOSS stage 2.

### 4.8. Statistical Analysis

Statistical analysis was performed using the SPSS (Statistical Package for Social Science) software (SPSS version 21.0, Chicago, IL, USA). Categorical variables were reported as count and percentage, while numerical variables were described as mean ± standard deviation (SD). Differences between stage groups in the continuous variables were assessed using the two-independent-sample *t*-test. The Cox proportional hazards regression model was used to assess the relative risk of comorbidities by EOSS stage. *p* values ≤ 0.05 were considered statistically significant.

## 5. Conclusions

Individuals with obesity classified in more advanced stages of EOSS had lower serum concentrations of 25(OH)D and a worse metabolic profile.

Considering that EOSS is more in line with the multifactorial aspects of obesity, the results found may support integrative interventions in obesity control, including monitoring the nutritional status of vitamin D and its relationship with metabolic changes.

## Figures and Tables

**Table 1:** Percentage of incidence of metabolic changes and comorbidities present in the sample according to the stages of the Edmonton Obesity Staging System (EOSS).

Metabolic Changes and Comorbidities	EOSS 0 *n* = 3		EOSS 1 *n* = 50		EOSS 2 *n* = 142		EOSS 3 *n* = 31		TOTAL
	*n* (%)	*p*	*n* (%)	*p*	*n* (%)	*p*	*n* (%)	*p*	*n*
IR	0 (0.0%)	0.202	36 (20.5%)	0.147	115 (65.3%)	0.127	25 (14.2%)	0.887	176
AH	0 (0.0%)	*0.002*	0 (0.0%)	*≤0.001*	133 (81.6%)	*≤0.001*	30 (18.4%)	*≤0.001*	163
MS	0 (0.0%)	0.242	12 (7.6%)	*0.004*	118 (75.2%)	*≤0.001*	29 (18.5%)	*0.007*	159
T2DM	0 (0.0%)	0.331	0 (0.0%)	0.135	43 (86.0%)	*≤0.001*	7 (14.0%)	0.959	50
Hypertriglyceridemia	0 (0.0%)	0.111	15 (17.1%)	0.277	56 (63.6%)	0.401	17 (19.3%)	*0.026*	88
Hypercholesterolemia	0 (0.0%)	0.817	0 (0.0%)	0.335	2 (66.7%)	0.881	1 (33.3%)	0.340	3
MAFLD	0 (0.0%)	0.432	3 (6.0%)	0.065	19 (38.0%)	*0.008*	28 (56.0%)	*0.004*	50
Hyperuricemia	0 (0.0%)	0.739	17 (23.8%)	0.231	42 (57.5%)	0.112	14 (19.2%)	0.201	73

EOSS = Edmonton Obesity Staging System; IR = insulin resistance; AH = Arterial Hypertension; MS = Metabolic Syndrome; T2DM = Type 2 Diabetes Mellitus; MAFLD = Metabolic-Associated Fatty Liver Disease.

**Table 2:** Edmonton Obesity Staging System (EOSS) definitions [4].

EOSS Stage	Conceptual EOSS Definition	Features Definition
**0**	No apparent obesity-related risk factors, physical symptoms, psychopathology, functional limitations, and/or impairments of well-being.	No EOSS factors were reported.
**1**	Presence of obesity-related subclinical risk factors, mild physical symptoms, mild psychopathology, mild functional limitations, and/or impairment of well-being.	Any of the following: Glucose ≥ 100.1 mg/dL Cholesterol ≥ 193.3 mg/dL Triglycerides ≥ 150.6 mg/dL HDL-c ≤ 61.9 mg/dL LDL-c ≥ 127.6 mg/dL SBP ≥ 130 mmHg DBP ≥ 85 mmHg
**2**	Presence of established obesity-related chronic disease, moderate limitations in activities of daily living, and/or well-being.	Any of the following: Glucose ≥ 126.0 mg/dL Diagnosed type 2 diabetes or type 2 diabetes medication Cholesterol ≥ 232.0 mg/dL (diagnosed with hypercholesterolaemia) Triglycerides ≥ 194.9 mg/dL HDL-c ≤ 38.7 mg/dL LDL-c ≥ 158.5 mg/dL Diagnosed hyperlipidemia or hyperlipidemia medication SBP ≥ 140 mmHg DBP ≥ 90 mmHg Diagnosed hypertension or hypertension medication Sleep apnea Gout Arthritis Anxiety Atherosclerosis Fatty liver Congestive heart-failure medication Blood-thinner medication Depression
**3**	Established end-organ damage, significant psychopathology, significant functional limitations, and/or impairment of well-being.	Any of the following: Angina Heart attack Heart failure Thrombosis Coronary artery disease Coronary obstructive pulmonary disease Dyspnea Exercise dyspnea Coronary artery bypass-surgery Stroke
**4**	Severe (potentially end-stage) disabilities from obesity-related chronic diseases, disabling psychopathology, functional limitations, and/or impairment of well-being.	No data on these factors available to evaluate this stage.

HDL-c: high density lipoprotein cholesterol; LDL-c: low density lipoprotein cholesterol; SBP: systolic blood pressure; DBP: diastolic blood pressure.

## Data Availability

The data used to support the findings of this study are available from the corresponding author upon request.
